# Oscillating paramagnetic Meissner effect and Berezinskii-Kosterlitz-Thouless transition in underdoped Bi_2_Sr_2_CaCu_2_O_8+δ_

**DOI:** 10.1093/nsr/nwad249

**Published:** 2023-09-18

**Authors:** Shiyuan Wang, Yijun Yu, Jinxiang Hao, Keyi Liang, Bingke Xiang, Jinjiang Zhu, Yishi Lin, Yinping Pan, Genda Gu, Kenji Watanabe, Takashi Taniguchi, Yang Qi, Yuanbo Zhang, Yihua Wang

**Affiliations:** State Key Laboratory of Surface Physics and Department of Physics, Fudan University, Shanghai 200433, China; State Key Laboratory of Surface Physics and Department of Physics, Fudan University, Shanghai 200433, China; State Key Laboratory of Surface Physics and Department of Physics, Fudan University, Shanghai 200433, China; State Key Laboratory of Surface Physics and Department of Physics, Fudan University, Shanghai 200433, China; State Key Laboratory of Surface Physics and Department of Physics, Fudan University, Shanghai 200433, China; State Key Laboratory of Surface Physics and Department of Physics, Fudan University, Shanghai 200433, China; State Key Laboratory of Surface Physics and Department of Physics, Fudan University, Shanghai 200433, China; State Key Laboratory of Surface Physics and Department of Physics, Fudan University, Shanghai 200433, China; Condensed Matter Physics and Materials Science Department, Brookhaven National Laboratory, Upton, NY 11973, USA; Research Center for Functional Materials, National Institute for Materials Science, Tsukuba 305-0044, Japan; International Center for Materials Nanoarchitectonics, National Institute for Materials Science, Tsukuba 305-0044, Japan; State Key Laboratory of Surface Physics and Department of Physics, Fudan University, Shanghai 200433, China; State Key Laboratory of Surface Physics and Department of Physics, Fudan University, Shanghai 200433, China; Shanghai Research Center for Quantum Sciences, Shanghai 201315, China; State Key Laboratory of Surface Physics and Department of Physics, Fudan University, Shanghai 200433, China; Shanghai Research Center for Quantum Sciences, Shanghai 201315, China

**Keywords:** cuprate superconductor, monolayer, Berezinskii-Kosterlitz-Thouless transition, scanning SQUID, paramagnetic Meissner effect

## Abstract

Superconducting phase transitions in two dimensions lie beyond the description of the Ginzburg-Landau symmetry-breaking paradigm for three-dimensional superconductors. They are Berezinskii-Kosterlitz-Thouless (BKT) transitions of paired-electron condensate driven by the unbinding of topological excitations, i.e. vortices. The recently discovered monolayers of layered high-transition-temperature (${{{T}}}_{{\rm C}}$) cuprate superconductor Bi_2_Sr_2_CaCu_2_O_8+δ_ (Bi2212) meant that this 2D superconductor promised to be ideal for the study of unconventional superconductivity. But inhomogeneity posed challenges for distinguishing BKT physics from charge correlations in this material. Here, we utilize the phase sensitivity of scanning superconducting quantum interference device microscopy susceptometry to image the local magnetic response of underdoped Bi2212 from the monolayer to the bulk throughout its phase transition. The monolayer segregates into domains with independent phases at elevated temperatures below ${{{T}}}_{{\rm C}}$. Within a single domain, we find that the susceptibility oscillates with flux between diamagnetism and paramagnetism in a Fraunhofer-like pattern up to ${{{T}}}_{{\rm C}}$. The finite modulation period, as well as the broadening of the peaks when approaching ${{{T}}}_{{\rm C}}$ from below, suggests well-defined vortices that are increasingly screened by the dissociation of vortex-antivortex plasma through a BKT transition. In the multilayers, the susceptibility oscillation differs in a small temperature regime below ${{{T}}}_{{\rm C}}$, consistent with a dimensional crossover led by interlayer coupling. Serving as strong evidence for BKT transition in the bulk, we observe a sharp jump in phase stiffness and paramagnetism at small fields just below ${{{T}}}_{{\rm C}}$. These results unify the superconducting phase transitions from the monolayer to the bulk underdoped Bi2212, and can be collectively referred to as the BKT transition with interlayer coupling.

## Teaser

This study used scanning SQUID susceptometry to observe oscillating paramagnetic Meissner effect in monolayer Bi2212 near its critical temperature, which served as a signature of Berezinskii-Kosterlitz-Thouless transition in this compound.

The Mermin-Wagner theorem [1] forbids long-range order at a finite temperature in a 2D system with continuous symmetry and short-range interactions. However, a phase transition of infinite order—the famed Berezinskii-Kosterlitz-Thouless (BKT) transition—into a phase without true long-range order is allowed [2–6]. In terms of superconducting systems, indirect signs of BKT transitions were observed in the charge transport of 2D arrays of Josephson junctions [7], ultrathin conventional superconductors [8,9] and superconducting interfaces [10]. Commonly regarded as the direct signature of the BKT transition, a discontinuity in superfluid phase stiffness, inevitably broadening to a continuous slope due to the finite sample size of a realistic 2D film, was observed by mutual inductance in YBa_2_Cu_3_O_7−_*_δ_* films [11]. A BKT transition is driven by the unbinding of vortex-antivortex pairs in a paired-electron condensate, which implies the existence of well-defined vortices, with a finite vortex core size at the transition temperature. This is different from a superconducting phase transition in 3D where the coherence length diverges and the vortex is no longer well-defined. As a result, in conventional 2D superconductors the BKT transition appears at temperatures below their Bardeen-Cooper-Schrieffer (BCS) pairing temperature, which corresponds to its bulk ${{{T}}}_{{\rm C}}$ [12].

The recently discovered monolayer Bi_2_Sr_2_CaCu_2_O_8+δ_ (Bi2212) showed very similar ${T}_{\mathrm{C}}$ and other electronic properties to those of the bulk [13], making it an ideal platform for studying how BKT physics evolves from the 2D limit to quasi-2D scenarios with interlayer coupling. The absence of any sign of BKT transition in the charge transport further confounded the mystery surrounding the highly debated pseudogap regime above the superconducting order regardless of sample thickness [6]. The ubiquitous emergent electronic orders have been known to be on the same energy scale as Cooper pairing in underdoped cuprates [14–17], which tends to cloud spectroscopic and thermodynamic discontinuities at the phase transition [18,19]. Earlier Nernst [20] and torque magnetization measurements [21] on bulk crystals showed evidence of vortex excitation in the pseudogap regime under strong magnetic field, suggesting preformed pairs above ${T}_{\mathrm{C}}$ [22]. However, these volumetric techniques are neither able to measure samples without a field nor sensitive enough for 2D materials. As a result, the nature of the superconducting transition in underdoped Bi2212 under different lattice dimensionality, and its relation with the pseudogap regime, remain unknown.

The spatial inhomogeneity of ultrathin Bi2212 [13,23] and the complication of the charge ordering demand highly sensitive magnetic imaging techniques for the study of the phase transition. To visualize the BKT transition, we employ scanning superconducting quantum interference device (sSQUID) microscopy [24–32], which has a high flux sensitivity without applying an external magnetic field. Our nano-SQUID device involves a nano-fabricated chip that integrates a 2-micron-diameter pickup loop into a two-junction SQUID that converts the flux through the loop ($\Phi $) into a voltage signal [33]. The pickup loops of our nano-SQUID are in a gradiometric design so that flux due to a uniform external field through both loops is cancelled out and the measured flux signal is strictly from the sample. In addition, we use mu-metal to shield the Earth's magnetic field and a home-wound coil to compensate for any residual field, allowing the sample to be measured in a true zero-field environment. By passing an alternating current (${I}_F$) through the field coil, we obtain the real part of the AC susceptibility (χ′) by demodulating the in-phase component from the flux signal in the pickup loop [34]. We thermally isolate our nano-SQUID from the sample so that the sample temperature can be independently raised to 200 K without introducing additional noise to the nano-SQUID, which is thermalized to 4.6 K [29]. Maintaining a low-noise environment for the sSQUID measurement over a large temperature range in a well-controlled magnetic field is critical for the investigation of the phase transition of cuprate high-temperature superconductors in the 2D limit.

**Figure 1.**
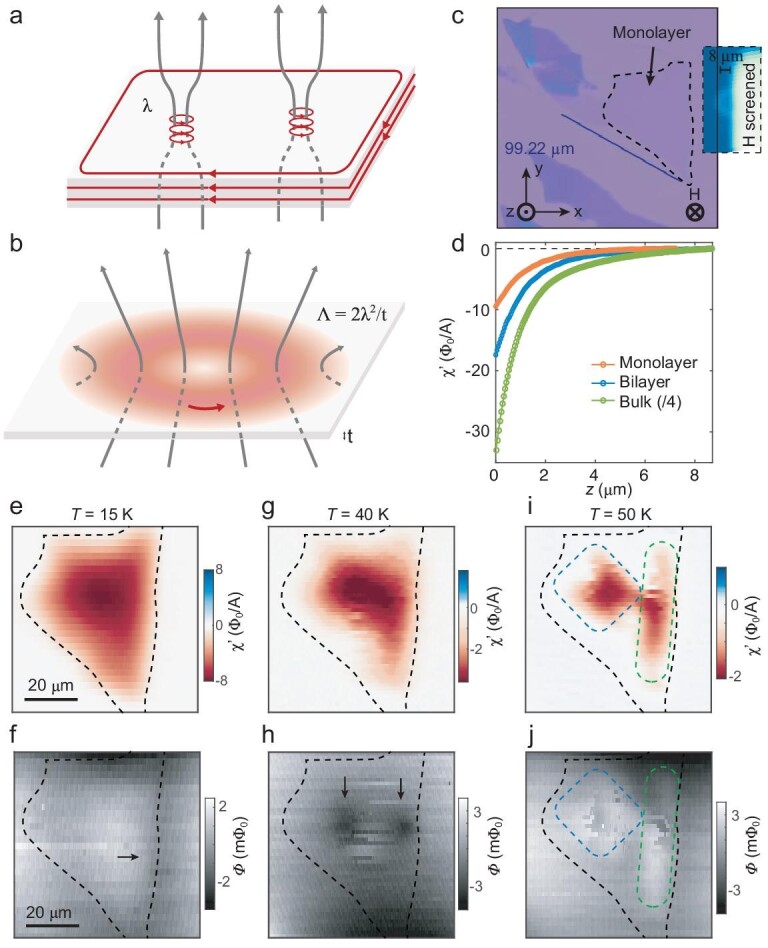
sSQUID magnetometry and susceptometry of an underdoped Bi2212 monolayer at various temperatures. (a and b) Illustrations of the vortices in a bulk and a 2D superconductor, respectively. The respective characteristic length scales of the vortices are the London penetration depth $\lambda $ and the Pearl length ${\mathrm{\Lambda }} = 2{\lambda }^2/t$, where *t* is the thickness of the 2D superconductor. The gray lines indicate the flux lines and the red lines represent the supercurrent around the vortices. (c) Optical image of the monolayer sample. Inset: susceptometry image of a nano-SQUID head showing the orientation of the field coil and the screening layer made of Nb in the same scale as the optical image of the monolayer sample. Only the area of the sample to the left of the screening layer is subject to an out-of-plane field *H*. (d) Susceptibility approach curves of the monolayer (orange), bilayer (blue) and bulk (green) samples. Diamagnetism corresponds to $\chi ^{\prime} < 0$. (e and f) Susceptometry and magnetometry images of the monolayer at 15 K, respectively, with *H* = 0.17 G. The shape of the superconducting region is traced out following the dashed line in (c). The arrow in (f) points to the edge contrast due to Meissner screening. (g and h, and i and j) Corresponding images at 40 K and 50 K, respectively. The discontinuous jump in magnetometry is a result of sporadic vortex motion intrinsic to the vortex glass state at this temperature and field regime. The blue and green dashed boxes in (i) and (j) roughly outline two domains in the interior of the sample with ${T}_{\mathrm{C}} = 64\ {\mathrm{K}}$. The arrows in (h) point to the flux contrast from Meissner current loops circling the boundary of these two domains. The exterior of the sample forms a separate domain with a lower ${T}_{\mathrm{C}} = 52\ {\mathrm{K}}$ due to loss of oxygen.

The magnetic behavior in the superconducting state of a conventional thin superconductor is already quite different from the bulk. Because of the Meissner effect, the magnetic field is screened out of a bulk superconductor except around vortices, which have a lateral span of London penetration depth ${\boldsymbol{\lambda }}$ [35–37] (Fig. 1a). Diamagnetism is much weaker in thin superconductors when sample thickness ${\boldsymbol{t}} \ll {\boldsymbol{\lambda }}$, allowing almost unhindered penetration of the magnetic field throughout the entire sample (Fig. 1b). The in-plane penetration depth ${\boldsymbol{\lambda }}$ in the bulk is replaced by the Pearl length ${{\bf \Lambda }} = \frac{{2{{\boldsymbol{\lambda }}}^2}}{{\boldsymbol{t}}}$ [32,38], which determines the size of Pearl vortices and Meissner current in thin superconductors [12]. ${{\bf \Lambda }}$ is significantly larger than ${\boldsymbol{\lambda }}$ so that the magnetic features in a 2D superconductor are much more spread out than in a 3D one. Moreover, the inverse Pearl length $1/{{\bf \Lambda }}$ is directly related to the superfluid density and phase stiffness, which are fundamental parameters in BKT physics. (The length scale for the variation of superfluid density, still determined by the coherence length, is not affected by thickness.)

To image the magnetic features in the superconducting state of ultrathin Bi2212, we exfoliated optimally doped Bi2212 single crystals. We followed the exfoliation technique described previously [13] to obtain thin flake samples of various thicknesses (Fig. 1c). The bulk ${T}_{\mathrm{C}}$ of Bi2212 is 88.2 K, as determined by both volumetric magnetometry and sSQUID susceptometry under zero magnetic field (supplementary online material). The approach curves on the thin flakes at 10 K can be well fitted with the model of a thin large diamagnetic disk (Fig. 1d) [39], which gives ${\mathrm{\Lambda }} = $ 171 μm for the monolayer (supplementary online material). Using $t = $ 1.5 nm for the monolayer, we obtain $\lambda = $ 358 nm, comparable to the in-plane penetration depth of the bulk Bi2212 with slight under-doping [40]. The deviation from optimal doping is probably due to loss of oxygen occurring after exfoliation and before an hexagonal-boron-nitride (h-BN) capping layer completely covers the monolayer sample (supplementary online material). This results in a loss of superconductivity of the left corner of the sample and reduces the ${T}_{\mathrm{C}}$ of the rest compared to the bulk ${T}_{\mathrm{C}}$ (Fig. 1e). Since ${\mathrm{\Lambda }}$ is larger than the size of the monolayer, there is no visible vortex in the interior of the monolayer under an out-of-plane field *H* = 0.17 G. The weak magnetic contrast at the edge, which has negative contrast relative to *H* just inside the boundary (Fig. 1f), suggests it is from a Meissner current.

The spatial variation of both the magnetization and susceptibility becomes much less uniform as the temperature increases. Even though χ′ (Fig. 1g) is qualitatively similar to its low temperature state, the Meissner current surrounding the whole sample shrinks and separates into two loops at 40 K (Fig. 1h). Since the diamagnetism at 40 K is much weaker than that at 15 K (Fig. 1e), meaning a much larger ${\mathrm{\Lambda }}$, the smaller and segregated flux feature can only be the result of the formation of domains of different superconducting phases. The sporadic jumps along the fast-axis of the magnetometry image (Fig. 1h), only appearing on the sample and also present in the bulk (supplementary online material), are due to free vortices quickly moving past the nano-SQUID during its slow scanning motion. Such a signal is intrinsic to Bi2212 at this temperature regime and is likely to be the result of its strongly layered structure [41] and weak pinning. The domains become more distinctive at 50 K in both χ′ and magnetometry images (Fig. 1f and j). The areas sandwiched between the two domains show diminishing diamagnetism as they have a lower ${T}_{\mathrm{C}} = 52$ K (defined by the temperature above which no diamagnetic feature can be observed). These two weakly superconducting domains disrupt the phase coherence between the left and right domains, which have ${T}_{\mathrm{C}} = 64$ K. The existence of domains can prevent bulk probes from resolving phase-coherent processes within the domains.

**Figure 2.**
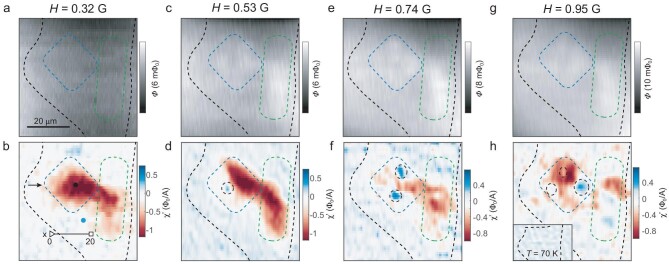
Appearance of paramagnetic regions under a small external magnetic field at elevated temperatures. (a–h) Magnetometry and susceptometry images for the monolayer sample shown in Fig. 1 obtained at 60 K, and various external field *H* as indicted on the panels. The dashed boxes roughly delineate the boundaries of the two interior domains. The paramagnetic Meissner effect appears as blue patches (dark dashed circles) in susceptometry images, which return to being diamagnetic with increasing field. (h inset) Susceptometry obtained at 70 K, showing the absence of either diamagnetism or paramagnetism.

At even higher temperatures, the susceptometry images become rapidly dependent on the magnetic field (Fig. 2). The overall diamagnetic signal is weaker at higher fields because an increased screening current costs free energy and reduces the superfluid density. (Alternatively, for 2D superconductors, this can be understood as field-induced vortices reducing the superfluid density.) Magnetometry does not show noticeable Meissner current (Fig. 2a) under a relatively small *H* = 0.32 G at 60 K, but the diamagnetic region shrinks and a small area of paramagnetic region occurs on the top (Fig. 2b). At *H* = 0.53 G, the contrast is enhanced a little in magnetometry (Fig. 2c). The paramagnetic region on the top turns diamagnetic, but another paramagnetic area occurs on the left side of the sample (Fig. 2d). As *H* increases further to 0.74 G, there is no qualitative change in the magnetometry image (Fig. 2e) but the left side reverts to being weakly diamagnetic again with development of other paramagnetic areas in the middle (Fig. 2f). The magnetometry image at *H* = 0.95 G (Fig. 2g) is similar to the one at *H* = 0.74 G, but the susceptometry is different and the paramagnetic pattern seems more random (Fig. 2h). The overall diamagnetic signal is weaker at higher fields than lower ones. The weak and similar magnetometry signal rules out the possibility that the paramagnetic signal in susceptometry is from cross-talk between the two channels. The reappearance of diamagnetism at the same location as the field increases that the sample is still in a superconducting state despite the reduced superfluid density under these fields. Furthermore, there is neither diamagnetism nor paramagnetism above ${T}_{\mathrm{C}}$ (Fig. 2h, inset).

Such paramagnetism in the superconducting state is reminiscent of the paramagnetic Meissner effect (PME, also called the Wohlleben effect), which was observed several decades ago in polycrystalline Bi2212 bulk samples by cooling under small magnetic fields [42,43]. The original explanation, which relied on *d*-wave pairing forming π-junctions across grains of different crystal orientation [44], was debated. It was later found that the PME might also occur in conventional mesoscopic superconductors [45] and the surface states [46] of odd-frequency superconductors [47–49]. Since our Bi2212 sample is single-crystalline, the domain boundaries are unlikely to form π-junctions regardless of pairing symmetry. Furthermore, the PME occurs inside the domain rather than on its boundary. As we shall show, our observation of sharp paramagnetic susceptibility in the Bi2212 monolayer is naturally interpreted in terms of the Coulomb plasma analogy first introduced by Kosterlitz and Thouless [3–5]. Based on this picture, vortex-antivortex pairs in a finite system interact with the free vortices introduced by the magnetic field, just like the Coulomb interaction in a 2D plasma. And the paramagnetic susceptibility reflects the vortex fugacity in a similar sense to the conductance into a quantum dot [50].

**Figure 3.**
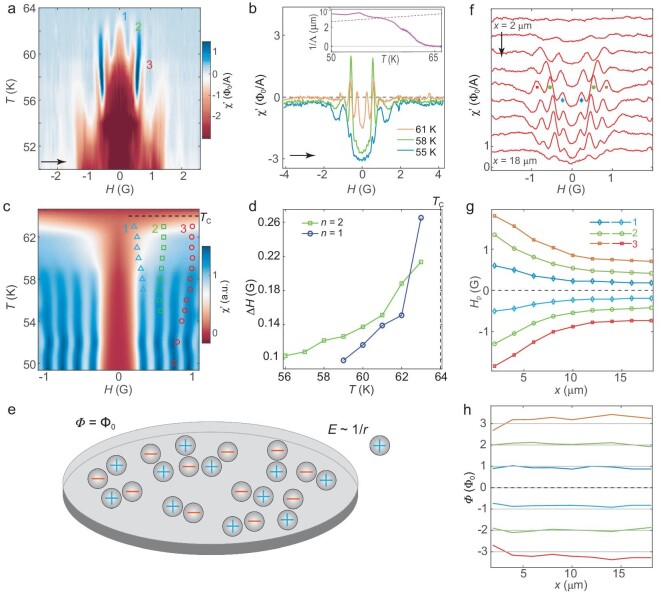
Oscillating paramagnetic susceptibility in the monolayer as a function of external field. The data are obtained on the diamond-shaped domain. (a and b) ${\mathrm{\chi }}^{\prime}( {T,H} )$ of the monolayer sample taken at the point shown in Fig. 2b. The data are obtained by sweeping the field at different *T*. The *T* axis is interpolated to generate the image in (a). Three representative temperatures of ${\mathrm{\chi }}^{\prime}( H )$ are shown in (b). Arrows indicate the direction of the sweep. The three lowest-order paramagnetic peaks are labeled by the orders ‘1’, ‘2’ and ‘3’. Inset: the inverse of the Pearl length, $1/{\mathrm{\Lambda }}( T )$, obtained from the diamagnetic susceptibility at zero field in (a). The diamagnetic data in the range of 60–66 K (inset) are from a fine temperature sweep at zero field (supplementary online material). The interception of the dashed straight line with $1/{\mathrm{\Lambda }}( T )$ loosely assigns a BKT crossover temperature (see text). (c) Simulated $\chi ( {T,H} )^{\prime}$ for the data in (a) (see text for details). The Meissner screening is ignored in the simulation and thus there is no diamagnetism in the susceptibility. Open symbols: peak position (${H}_p$) as a function of *T* extracted from (a). The three lowest-order paramagnetic peaks are represented by blue, green and red symbols, respectively. (d) Half-width of the first two paramagnetic peaks obtained by fitting the ${\mathrm{\chi }}^{\prime}( H )$ curves in (a). (e) Illustration of the interaction of a free vortex (charge outside the island) and 2D vortex-antivortex plasma in a finite system, in analogy with the 2D Coulomb gas model. (f) ${\mathrm{\chi }}^{\prime}( H )$ curves obtained at 60 K at different displacement *x* of the nano-SQUID tip over the monolayer sample along the arrow shown in Fig. 2b. The curves with larger *x* are shifted downward proportionally. (g) ${H}_p( x )$ extracted from (f). (h) ${H}_p( x )$ from (g) plotted in units of flux quantum. This is obtained by integrating the field over the exposed area of the domain (see Fig. 1c and text).

In order to elucidate the origin of the PME in the monolayer Bi2212, we measure susceptibility as a function of magnetic field and temperature (*T*) at a fixed location within a domain. We pick the middle point of the diamond-shaped domain of the monolayer sample (as shown by the black dot in Fig. 2b). The susceptibility obtained by sweeping *H* at various *T* (Fig. 3a) shows the most pronounced paramagnetism between 50 K and ${T}_{\mathrm{C}}$ = 64 K. Other than the spikes, the field sweep χ′ curves are typical of a type II superconductor [51]: a flat diamagnetic bottom at low field, which starts to increase at lower critical field (${H}_{c1}$) around 0.5 G (Fig. 3b). χ′ levels off to values slightly below zero at higher fields, which are four orders of magnitude smaller than the upper critical field, at which the superfluid density vanishes ($>\! 4.6\ {\rm T}$ at 61 K for ${T}_{\mathrm{C}}$ = 64 K). We note here that the ${H}_{c1}$, defined as the field at which the first vortex enters the sample, is determined by different physics in 2D and in 3D. In bulk superconductors, ${H}_{c1}$ is proportional to the surface energy density at the superconducting-normal boundary and is independent of sample size [35]. This is not the case here given the large Pearl length. ${H}_{c1}$ is close to the first PME peak and the reason will become clear later. The PME disappears above ${T}_{\mathrm{C}}$ (Fig. S4), which again shows that it is related to superconductivity.

The PME appears as several symmetric spikes at fields ${H}_p$ overlayed on top of the diamagnetic background in the field sweep curves (Fig. 3b), which slowly shift with temperature (Fig. 3a). The spikes that are closest to zero field (labeled as ‘1’) disappear below 59 K. The higher-order spikes (second and third are labeled as ‘2’ and ‘3’, respectively) persist to lower temperatures. The oscillating PME with *H* is clearly not due to vortex melting [51,52]. Vortex melting typically only exhibits a singular paramagnetic peak at a large enough field to generate high vortex density for the melting transition [53]. When temperature is continuously varied rather than the field, the *H-T* diagram obtained from such ‘field-cooling’ cycles shows different peak amplitude but the same peak position in field ${H}_p$ (Fig. S4). The ${H}_p$ is also independent of the modulation field frequency or amplitude if it is small, but they are suppressed when the modulation amplitude is comparable or larger than the peak spacing in *H* (supplementary online material). These characterizations rule out a resonance artifact in the AC susceptibility and provide further evidence that the oscillating PME is an intrinsic response of the Bi2212 monolayer under an external field.

The temperature dependence of the PME peaks distinguishes this phenomenon from conventional vortex dynamics in a superconductor. The disappearance of the PME peaks is at the same temperature as the disappearance of diamagnetism at zero field, i.e. ${T}_{\mathrm{C}}$ = 64 K. The ${H}_p$’s of the lowest three peaks (Fig. 3c, open symbols) do not go down to zero at ${T}_{\mathrm{C}}$, manifesting robust phase coherence all the way to the transition. This is fundamentally different from the paramagnetic effect associated with conventional vortex dynamics [30,45,54], which does not survive to ${T}_{\mathrm{C}}$ due to diverging vortex cores. In sSQUID susceptometry of 2D superconductors, Pearl length ${\mathrm{\Lambda }}$ is inversely proportional to the real part of AC susceptibility ${\mathrm{\chi }}^{\prime}$ (supplementary online material). Converting the zero field ${\mathrm{\chi }}^{\prime}( T )$ to 1/${\mathrm{\Lambda }}( T )$ (Fig. 3b inset), we find that the phase stiffness continuously changes with temperature across ${T}_{\mathrm{C}}$. The interception $1/{\mathrm{\Lambda }}( {{T}_{{\mathrm{BKT}}}} ) = \frac{{4\pi {\mu }_0}}{{{\mathrm{\Phi }}_0^2}}k{T}_{{\mathrm{BKT}}}$, where ${\mu }_0$ is the vacuum permeability, ${{\mathrm{\Phi }}}_0$ the flux quantum and *k* the Boltzmann constant, corresponds to the BKT temperature ${T}_{{\mathrm{BKT}}}$ in the thermodynamic limit. For a finite-sized domain in our case, the universal jump in the phase stiffness is smoothed out by finite-size effects [55] and the interception temperature merely provides an energy scale for the BKT crossover.

Despite the absence of the susceptibility jump, vortex-antivortex dissociation in a finite-sized 2D superconductor is manifested through the fluctuation of vortices. Such fluctuation is evident from the broadening of PME peaks. Both of the first two peaks broaden significantly as we approach ${T}_{\mathrm{C}}$ from below (Fig. 3d). (The third peak is so broad to start with that we do not get reliable width as temperature increases.) At ${T}_{\mathrm{C}} = $ 64 K, the peaks are totally merged together, consistent with divergent vortex fluctuation at the transition. The persistence of phase coherence at ${T}_{\mathrm{C}}$, and the increasing vortex fluctuation with increasing temperature, are both directly linked to a vortex-driven phase transition in a finite 2D superconductor. Below we explain our observation using the analogy of a Coulomb blockade [50] of a Coulomb plasma.

The connection between vortices in a 2D superconductor and 2D Coulomb gas is a consequence of the long-range ${\mathrm{ln}}( {r/\xi } )$ interaction between two Pearl vortices separated by a distance *r* being identical to the Coulomb interaction in 2D electrodynamics. Using this analogy, previous theoretical and numerical studies of an *infinite* 2D XY model under weak frustration did find paramagnetic susceptibility [56]. The magnetic field, due to the negligible screening in 2D, acts as a chemical potential for the Coulomb gas [57] inside our monolayer sample, which is essentially an island for the vortices considering their Pearl length (Fig. 3e). When this chemical potential exceeds the free energy cost of a vortex, a single vortex has a high probability of being excited and the field defines ${H}_{c1}$. If the island under consideration has no vortex/antivortex background, the entrance of the first vortex gives rise to a paramagnetic peak that is infinitely sharp. However, since we have a vortex-antivortex plasma, the PME peak is broadened due to the screening of the plasma, with the width of the peak reflecting the dielectric constant. Upon further increase of the magnetic field, additional vortices enter one-by-one when the chemical potential overcomes the expulsion energy of the vortices already on the island but screened by the vortex plasma. As ${T}_{\mathrm{C}}$ is approached from below, the peaks remain at finite fields because the superfluid density renormalizes both the repulsive interaction and the chemical potential of the vortices, while the width of peaks diverges because of the complete screening of inter-vortex interaction (supplementary online material).

We perform a Monte Carlo simulation of the 2D Coulomb-gas model to compute the paramagnetic response of the vortices in a finite circular disk. The high symmetry of the geometry simplifies the computation although admittedly it does not match the actual shape of the sample. We find qualitative agreement on ${\mathrm{\chi }}^{\prime}( {T,H} )$ between the modeling (with only two fitting parameters) and the experiment (Fig. 3c). The extracted first two PME peaks evolving with temperature also agree with those of the experiment (Fig. 3c, blue and green symbols). The main discrepancy occurs for the third peak where the modeling underestimates the peaks (Fig. 3c, red symbols). This is likely caused by our crude approximation of the shape of the sample, which affects the supercurrent distributions and energy of the vortices, and consequently the peak positions, by an order-1 numerical factor. Nevertheless, the modeling captures the most striking feature of the experiment: the ${H}_p$’s are finite at ${T}_{\mathrm{C}}$ while the peaks broaden and disappear (Fig. 3c and d).

Now that the connection between the oscillating PME and BKT physics is established, we turn to the spatial variation of oscillating PME. We obtain ${\mathrm{\chi }}^{\prime}( H )$ curves at various horizontal positions *x* across the left domain (indicated by the arrow in Fig. 2b) of the monolayer sample at 60 K. The lowest three spikes in the *H-T* sweep (Fig. 3a) are still clearly distinguishable at all of the *x* positions except for the one at the left edge (Fig. 3f). All the spikes shift towards smaller *H* in a uniform fashion as *x* increases (Fig. 3g). This can be well understood when we consider field screening from the nano-SQUID (Fig. 1c, inset): the sample area on the right side of the pickup loop is completely shielded from the small external field we apply and only the area to the left and underneath the pickup loop is subjected to *H*. Normally this would not lead to any observable effect as only the field on the sample directly underneath the pickup loop matters. Here, however, it is the total flux threading the sample rather than the field that determines the susceptibility. As the nano-SQUID moves to larger *x*, the exposed area increases, and a smaller *H* is needed to maintain the same flux. This point can be best shown by multiplying ${H}_p$ and the exposed area to obtain the total flux through the sample as a function of *x* (Fig. 3h). Since diamagnetism of the monolayer sample at this temperature region is quite weak, as evident from the magnetometry, Meissner screening from the sample is ignored in this estimate. Given the uncertainty in the domain size, we normalize the lowest order peak in flux to the flux quantum. Besides the uncertainty in its area, a vortex in the monolayer will not in general carry a complete flux quantum ${{\mathrm{\Phi }}}_0$ due to the large Pearl length of the vortex. The position-independence and the approximately equal spacing between the lowest three peaks (Fig. 3h) is consistent with our Coulomb blockade model of interacting vortices (Fig. 3e).

Using the ${H}_p( x )$ relation of the three lowest-order spikes (Fig. 3g), we can now understand the seemingly random paramagnetic features we observed in the susceptometry images at 60 K (Fig. 2). Since the flux through the domain at a constant *H* increases as the nano-SQUID is moved from left to right, the paramagnetic patches in each image do not correspond to vortex patterns (see Fig. S3 for an illustration). Rather, the paramagnetic signal appears whenever an exposed area of the domain carries a quantized flux. Thus, the total number of paramagnetic patches within the domain at a specific *H* represents the number of vortices penetrating the entire domain when the nano-SQUID lies outside the right boundary of the domain. By counting the number of paramagnetic patches, we find that one vortex has penetrated the domain at *H* = 0.53 G (Fig. 2d), two at *H* = 0.74 G (Fig. 2f) and three at *H* = 0.95 G (Fig. 2h), respectively.

The scale of the domain size *R ∼*10 μm over which the coherent oscillation occurs is a thousand times larger than the coherence length of Bi2212 (∼16 nm even at 63 K for ${T}_{\mathrm{C}}$ = 64 K using BCS theory). This length scale also distinguishes our observations from the oscillating PME seen in a conventional mesoscopic superconductor [45,54], which requires a sample size close to its coherence length. By a similar consideration of the disparity in scales, the oscillating PME in monolayer Bi2212 cannot be due to the Little-Parks effect [58] because the change in critical temperature would have been unnoticeable: $\frac{{\Delta {T}_{\mathrm{C}}}}{{{T}_{\mathrm{C}}}} = 0.55{( {\frac{{{\xi }_0}}{R}} )}^2$, where ${\xi }_0$ is zero-temperature coherence length. As a control experiment, ultrathin NbSe_2_ flakes, which have a very similar ${\xi }_0$ and penetration depth, do not show any PME over a similar normalized temperature ($T/{T}_{\mathrm{C}})$ and field range (Fig. S11). This is not surprising because the vortex core size is divergent and phase fluctuation is thermally induced in a conventional superconducting phase transition, which suppresses phase coherence. Another factor pertaining to Bi2212 is its much higher ${T}_{\mathrm{C}}$ and larger pairing gap, which facilitates the proliferation of phase fluctuations without diminishing the amplitude of the order parameter.

**Figure 4.**
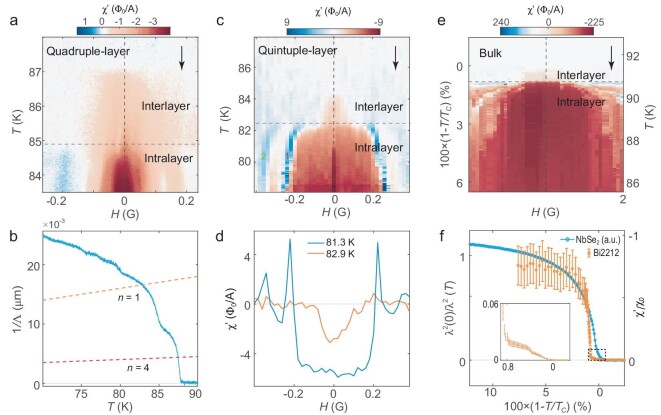
Paramagnetic Meissner effect in multilayers and a bulk sample. (a) ${\mathrm{\chi }}^{\prime}( {T,H} )$ in a quadruple-layer sample. Denoted by the gray dashed lines at ${T}_{\mathrm{P}}$, the susceptibility in the multilayers separates into two temperature regimes where intralayer and interlayer phase coherence is established. (b) $1/{\mathrm{\Lambda }}( T )$ of the quadruple-layer sample obtained from ${\mathrm{\chi }}^{\prime}( T )$ at zero field, taken over an extended temperature range. (c) ${\mathrm{\chi }}^{\prime}( {T,H} )$ in a quintuple-layer sample obtained by sweeping *T* at fixed *H*. (d) ${\mathrm{\chi }}^{\prime}( H )$ at two temperatures (replotted from (c)) above and below ${T}_{\mathrm{P}}$ respectively. (e) ${\mathrm{\chi }}^{\prime}( {T,H} )$ in a bulk Bi2212. Note that the ‘noisy’ diamagnetic signal in the Bi2212 bulk under finite *H* is absent above ${T}_{\mathrm{C}}$ and is therefore intrinsic to its superconductivity. (f) Normalized superfluid density ${\lambda }^2( 0 )/{\lambda }^2( T )$ (orange dots) of a bulk sample extracted from the fittings of susceptibility touch-down curves under zero field. The inset is a zoom-in of the temperature regime between ${T}_C$ and${\mathrm{\ }}{T}_P$. Blue curve: ${\mathrm{\chi }}^{\prime}( T )$ under zero field normalized by its susceptibility at base temperature ${{\mathrm{\chi }}}_0$ of a NbSe_2_ bulk control sample (${T}_{\mathrm{C}} = 6.6$ K). The sharp jump of superfluid density at ${T}_{\mathrm{C}}$ suggests BKT transition in the bulk Bi2212. There is no interpolation in any of the ${\mathrm{\chi }}^{\prime}( {T,H} )$ images (a), (c) and (e). Additional data on these samples are available in supplementary online material.

Besides the monolayer, the oscillating PME occurs generally in the ultrathin Bi2212 samples we study. For example, a quadruple-layer sample (${T}_{\mathrm{C}} = 87$ K) similarly exhibits paramagnetic peaks (Fig. 4a). The jump in phase stiffness is also reduced to a continuous slope below ${T}_{\mathrm{C}}$ due to the finite size effect (Fig. 4b). However, the susceptibility in multilayer samples has two noticeable differences compared to the monolayer: the PME oscillation in the multilayers is only present below a temperature ${T}_{\mathrm{P}} < {T}_{\mathrm{C}}$ (Fig. 4a) and the oscillating PME occurs simultaneously with a steeper rise in phase stiffness (Fig. 4b) at ${T}_{\mathrm{P}}$. The PME of a different domain on this quadruple layer with slightly lower ${T}_{\mathrm{C}}$ (supplementary online material) and a quintuple-layer sample are similarly separated into two temperature regimes (Fig. 4c and d). The absence of oscillating PME in the temperature regime above ${T}_{\mathrm{P}}$ suggests it is not caused by disparate ${T}_{\mathrm{C}}$’s of the surface layer and the buried layers.

The absence of ${T}_{\mathrm{P}}$ in the monolayer suggests this additional characteristic temperature in the multilayers is related to interlayer coupling. Since Bi2212 has weak interlayer coupling relative to other cuprates, ${T}_{\mathrm{C}}$ still corresponds to the onset of intralayer phase coherence [59]. However, the large interlayer phase fluctuation of a quasi-2D system in the regime ${T}_{\mathrm{P}} < T < {T}_{\mathrm{C}}$ prevents out-of-plane phase coherence, so no oscillating PME is observed. In turn, the phase stiffness represents an arithmetic sum of the superfluid density of individual layers. When the interlayer phase coherence sets in at ${T}_{\mathrm{P}}$, the domain is fully phase coherent and oscillating PME develops. Because the superfluid can now move in unison across a thicker plane, the phase stiffness is boosted again [60].

In even thicker samples, inhomogeneous domains in different layers are more prominent, such that their contributions to the PME are less in phase. This is clearly the case in a 20-nm sample where the PME peaks occur at seemingly random fields (Fig. S9c). In a bulk sample, which is roughly 200 nm thick, the domains are even more ‘coarse grained’. As a result, the PME peaks do not show any oscillation with field and only a small range of temperature close to ${T}_{\mathrm{C}}$ exhibits an overall paramagnetic response (Fig. 4e), reminiscent of the original observation of PME in cuprates by volumetric magnetometry [42,43]. However, it is still unclear why the original observations were only on polycrystalline samples. Such PME is clearly absent from the bulk NbSe_2_ (${T}_{\mathrm{C}} = 6.6$ K) control sample (supplementary online material). The presence of PME at finite fields, but absence of clear oscillations, follows the trend in thicker samples (Fig. S9c). The ‘smearing out’ of the peaks suggests that it becomes difficult for domains of random sizes in different layers to interfere constructively as the layer number becomes large.

The temperature dependence of the phase stiffness in bulk Bi2212 and NbSe_2_ are also markedly different (Fig. 4f). We extract $\lambda $ of bulk Bi2212 from the approach curves obtained under zero field at each temperature point [39] and then obtain the normalized phase stiffness ${\lambda }^2( 0 )/{\lambda }^2( T )$ (supplementary online material). Both ${\lambda }^2( 0 )/{\lambda }^2( T )$ of bulk Bi2212 and ${\mathrm{\chi ^{\prime}}}$ of NbSe_2_ are normalized for analysis of superfluid jump behavior near critical temperature. While the latter shows a smooth power-law rise below ${T}_{\mathrm{C}}$ typical of a BCS superconductor, the ratio ${\lambda }^2( 0 )/{\lambda }^2( T )$ in Bi2212 shows a sharp jump rising to 85% of its peak magnitude within 0.15% of ${T}_{\mathrm{C}}$. The sharp jump in superfluid density at zero field is precisely what we expect for a BKT transition in the thermodynamic limit [12]. Interestingly, there is also a ‘foot’ feature with small but finite phase stiffness within a temperature region 0.8% of ${T}_{\mathrm{C}}$ above the sharp jump and below ${T}_{\mathrm{C}}$ (Fig. 4f inset). This behavior is consistent with that of multilayers where there is interlayer phase coherence (Fig. 4a, c and e), although over a reduced temperature window.

The oscillating PME we have observed in Bi2212 is expected to be a general feature of underdoped cuprate superconductors, since this family shares a common layered structure, with various degrees of interlayer coupling. Regardless of lattice dimensions, Cooper pairs formed at a very high temperature give rise to a condensate in which binding of free vortices facilitates the superconducting transition. Our results obtained under zero or low fields suggest that BKT physics is important for understanding the universal scaling between the superfluid density and ${T}_{\mathrm{C}}$ [22,61], the abnormal critical behavior of the specific heat of underdoped YBa_2_Cu_3_O_6+x_ [19] and the Nernst effect in the pseudo-gap phase [20].

In conclusion, we have observed PME oscillations in monolayer and few-layer Bi2212, which evolve to be continuous with the field in thick samples. The oscillation with total flux through a domain persists to ${T}_{\mathrm{C}}$ in the monolayer and serves as direct evidence of phase coherence and finite vortex cores, which are the topological defects driving the BKT transition. The broadening of the PME peaks when approaching ${T}_C$ from below indicates the increasing screening of intervortex interactions—a clear sign of the imminent dissociation of vortex-antivortex pairs at the BKT transition. Our observations of the multilayers and thick samples show that the superconducting transitions in underdoped Bi2212 are generalized BKT transitions with interlayer coupling. The oscillating PME represents a novel and direct signature of BKT physics in realistic 2D superconductors.

## Supplementary Material

nwad249_Supplemental_File

## Data Availability

The data that support the findings of this work are available from the corresponding authors upon reasonable request.
