# Supplementary material for: Oscillating paramagnetic Meissner effect and Berezinskii-Kosterlitz-Thouless transition in underdoped Bi2Sr2CaCu2O8+δ
Source: Natl Sci Rev. 2023 Sep 18;11(5):nwad249. doi: 10.1093/nsr/nwad249 (PMC10989300; doi:10.1093/nsr/nwad249)
Supplement: nwad249_Supplemental_File [file nwad249_supplemental_file.pdf]

Supplementary Materials for

"Oscillating paramagnetic Meissner effect and Berezinskii–Kosterlitz–Thouless transition in underdoped $Bi_2Sr_2CaCu_2O_{8+\delta}$"

This file includes:

I. **Materials and Methods**

II. **Supplementary Text**

III. **Figs. S1 to S12**

I. Materials and Methods

1. Fabrication and characterization of ultrathin samples

We follow the previous procedure to fabricate monolayer and multilayer Bi2212 and $NbSe_2$ flake samples. We determine the thickness of the prepared sample by atomic force microscopy (Fig. S1) and establish a calibration of the thickness and optical contrast. Since the monolayer and ultrathin samples for sSQUID measurements have to be capped by hBN immediately after fabrication, we use their optical image to determine the thickness.

The bulk crystal shows diamagnetic response from around 90 K as measured by a commercial vibrating sample magnetometer (Fig. S2a). This is consistent with the T_C measured by sSQUID of a thick flake sample (Fig. 3e) exfoliated from the sample ingot and is close to the optimal T_C of Bi2212. The diamagnetic moment reduces as a function of increasing external field (Fig. S2b). However, no paramagnetic signal is visible in such bulk measurements.

2. Scanning SQUID magnetometry and susceptometry

1) Scanning SQUID measurements are performed in a closed-cycle He-4 refrigerator. The SQUID sensors used for this study are scanning 2-junction SQUID susceptometers with two balanced pickup loops of 0.5 or 3 μm diameter in a gradiometric configuration, each surrounded by a one-turn field coils of 5 or 7.5 μm diameter. These devices are planarized throughout, which minimize the spacing between the pickup loop-field coil pair and the sample surface. Because the pickup loop is almost parallel to the sample surface, it is only sensitive to the local out-of-plane magnetic field. The spatial resolution is limited by both the size of the pickup loop and the height of it from the sample.

For the scanning SQUID measurements throughout this paper, we employ magnetometry simultaneously with susceptometry [27]. Magnetometry (Φ) is a direct-current (DC) measurement of flux through the pickup loop as a function of position and shows the intrinsic magnetization of the sample; susceptometry ($d\Phi/dI_F$) measures the inductive response of the sample through the pickup loop to a modulation current in the field coil I_F. Such alternate-current (AC) susceptibility is demodulated by a lock-in amplifier and its in-phase component is taken as the real part of the AC susceptibility (χ'). We typically use 200 ms time-constant for the demodulation, but changing it to 50 ms does not change the features in susceptometry. We balance the I_F flowing through the front and back field coils so that the background demodulated signal is minimized. For any remaining background susceptibility, we use the χ' taken on the substrate as the reference for zero.

The sample approach is carried out under height control by a capacitive method. Our nano-SQUID chip is attached to a long copper cantilever which forms a capacitor with another parallel copper plate. The capacitance on the order of pico-farads is read out by a homemade AC capacitance bridge placed close to the capacitive sensor. Once nano-SQUID touches down on the sample, a large capacitance change occurs and its vertical scanner position is recorded as the approach point. We then retract the vertical scanner by 1 micron and start scanning imaging at this constant height.

2) Variable temperature scanning SQUID measurement is challenging. Despite the thermal isolation, the nano-SQUID sensor's temperature still slightly changes with the change of sample temperature, which causes the lock point of the flux-locked loop to shift slightly. But AC flux signal such as magnetic susceptibility keeps stable despite the shift in the DC flux signal.

Large temperature variation may cause thermal expansion in the piezoelectric scanner. To examine its influence on sSQUID microscopy, we perform imaging on a meandering current pattern under different temperatures (Figs. S3a-c). An AC current with constant amplitude is applied to the circuit and we measure this current flux signal under different temperatures by

demodulating the AC component from the DC flux signal. The current flux signal from the meandering pattern shows no noticeable difference at 80 K (Fig. S3b) and 90 K (Fig. S3c), implying that under the spatial resolution of our probe, there is no observable in-plane shift within a temperature window of about 10 K, which is the typical range of our fine temperature sweeps.

The relative thermal expansion between the sample stage and the scanning head is another factor to correct for variable temperature measurement. To compensate for it, we first determine the approach points at each temperature (Fig. S3d). Then we perform the variable temperature measurements at the same scanner height (of 1 micron) with respect to the approach point at that temperature. A fixed-point current flux sweep as a function of temperature (Fig. S3e) demonstrates the validity of our vertical shift compensation. The current flux approach curve as a function of vertical offset is plotted simultaneously in Fig. S3e. By converting the current flux fluctuation (Fig. S3e, grey) to the current flux approach curve, we estimate a 80 nm overall shift under a 10 K range temperature sweep sequence.

3) We use a home-wound coil around our cryostat to apply an out-of-plane magnetic field *H*. We calibrate the magnetic field the coil generated by measuring a thick Nb film. Any remanent field at the sample position is compensated by this coil.

The magnetic shielding layer on the SQUID chip must be taken into consideration when the SQUID is scanning above the sample under a non-zero magnetic field. The relative position of SQUID and sample is shown in Fig. S4. Since the magnetic field *H* we apply to the sample is far below the critical field of niobium, the field only goes through the space outside the shielding layer and the field under the shield is still zero, paramagnetic signal appears when quantized flux go through the domain, at which moment the space outside the shield S_1 reaches some specific value satisfying $H * S_1 = \mathrm{n}\Phi_0$.

II. Supplementary Text

1. Estimate Pearl length and superfluid density from susceptometry

We use Pearl length Λ to characterize the diamagnetic length scale of a weakly diamagnetic film, whose thickness is much less than the London penetration depth. In scanning SQUID susceptometry of 2D superconductors, Λ is related to real part of the susceptibility χ' as [37]: $\chi'/\chi_s = -a/\Lambda(1 - \frac{2\bar{z}}{\sqrt{1+4\bar{z}^2}})$, where $\chi_s = 490\ \Phi_0/\mathrm{A}$ is the self-inductance, $a = 5$ μm is the radius of the field coil and $\bar{z} = z/a$. Fitting the touch-down curve as shown in Figure 1b using the above equation, we obtain Λ of the monolayer to be 171 μm.

Besides the relation mentioned in the main text, $1/\Lambda$ is also proportional to the sheet superfluid density $\rho_s^{2D} = \rho_s t$ following the relation [9]: $\rho_s^{2D} = \frac{2m_e^*}{\mu_0 e^2 \Lambda}$ where m_e^* and e are the effective mass and charge of the electron and μ_0 is the vacuum permeability. Due to the uncertainty of the experimental geometry, the $1/\Lambda$ we obtain from χ' also has a systematic error [30]. However, since we use the same z for the constant height measurements, this systematic error led to an overall scaling factor on $1/\Lambda$ independent of the temperature. The three-dimensional superfluid density of bulk samples determined from fitting the susceptibility approach curves will be discussed in Sec. 2.4.

2. Additional data on different samples

1) Monolayer Bi2212

Fig. S5 shows the χ'(*H, T*) of the monolayer obtained at the same location as Fig. 3a (black dot in Fig. 2b). Here, *T* is swept at *H*. The paramagnetic peaks disappear simultaneously with diamagnetic susceptibility at zero field above $T_C = 64$ K of this domain. Furthermore, the peak positions in *H* (H_p) of χ'(*H, T*) do not depend on whether *H* or *T* is swept (Fig. S5 and Fig. 3a).

However, the amplitude of the peaks does depend on the history, which can be seen from a much-reduced contrast of peak ‘2’ (Fig. S5).

The perimeter of the sample tends to lose more oxygen during the fabrication process and therefore has lower T_C than the interior. But we observe similarly sharp PME peaks at the blue point of the monolayer (Fig. 2b), which has a lower T_C = 52 K (Fig. S6a). However, there are more orders of the peaks visible (Fig. S6b) and the spacing between the peaks is smaller than that of the diamond-shaped domain (Fig. 3b). This suggests that the blue point is inside a domain with larger size than the diamond-shaped domain. As the overall size of the diamond-shaped domain is only a small fraction of the whole sample (Fig. 2b), we speculate that the exterior is composed of multiple domains which surrounded the interior domains.

Changing the modulation frequency in the low frequency regime does not affect H_p (Fig. S7a). This rules out the PME peaks as a coincidental resonance peak from the AC measurement. The amplitude of the peaks reduces with increasing frequency. Since the peaks only occurred when flux through the domain is close to quantization, increasing the modulation frequency smears out the point of the phase coherence and therefore reduces the amplitude of the peaks. This observation also suggests that the PME peaks will be even stronger in the zero-frequency limit.

The modulation amplitude dependence is consistent with our main conclusion. The H_p also does not depend on the modulation amplitude when the modulation current is less than 0.15 mA (Fig. S7b). However, when the current is over 0.4 mA the peaks are again smeared out (Fig. S7c). This can similarly be explained by the phase coherence condition being compromised as the modulation flux is comparable to the overall phase in the domain. The modulation amplitude dependence is against the scenario where the AC magnetic field generated by the modulation current wiggles the vortex [28], which would have made the paramagnetic signal stronger with increasing modulation amplitude. The vortex-wiggling scenario would also be insensitive to the variation of the field and would disappear far under T_C.

2) Quadruple-layer Bi2212

For a quadruple-layer Bi2212 sample (8 nm thick), we observe in the magnetometry image isolated vortices as well as flux contrast at the edge of the sample due to the circulating Meissner current after it is field cooled and measure under 117 mG at 10 K (Fig. S8). The large size of the vortices in the magnetometry image is a reflection of its Pearl length, estimated to be ~ 10 μm from the approach curve. The susceptibility is diamagnetic over the entire sample and no normal core is visible (Fig. S8b) as its coherence length of ~ 2 nm is 3 orders of magnitude smaller than the diameter of the pickup loop.

What is different is that single vortex can be trapped and resolved in the multi-layers. This allows us to further confirm a deep connection between the paramagnetic response and the quantized flux instead of the field. When there is no trapped vortex and only a very small field is applied (Fig. S8c), there is little paramagnetic region in χ' even at 82 K (Fig. S8d). However, after we perform a field cooling to trap a single vortex in the middle of the sample (Fig. S8e), paramagnetic contrast appears under zero applied field at the same location as the vortex (Fig. S8e). Again, such paramagnetic signal in susceptibility is not a result of the measurement of the spatially varying flux signal because much stronger flux gradient at lower temperatures did not lead to any paramagnetic signal. Rather, it is an intrinsic paramagnetic response of that particular domain when there is a vortex present.

At a different location on this quadruple-layer sample close to its middle (Fig. S8f), The T_C of this area is about 0.5 K lower than the one from the upper-right part of the sample (Fig. 4a). We observe a slightly different $\chi'(H, T)$ pattern (Fig. S9a) than the latter. Nevertheless, the qualitative feature remains the same that the oscillating PME separates into two different temperature regimes, above and below 84.9 K respectively. The zero-field $\chi'(T)$, proportional to the phase stiffness, also exhibits a kink at this temperature (Fig. S9b). These two features in the multilayers are fundamentally different from the behavior of the monolayer and suggest its origin from the interlayer coupling.

3) 20-nm Bi2212

As the sample gets even thicker, the variation of the size of the domains from each layer becomes more significant. This results in more sporadic appearance of the PME peaks as can be seen from the χ'(*H, T*) data of the 20-nm thick sample (Fig. S9c). The height of the peaks reduces and the width in *H* is broadened comparing with those of the thinner samples. This suggests that the contribution to susceptibility from each layer is not in phase due to the difference in domain size. Such a tendency continues with sample thickness and completely smears out the PME oscillations in the bulk sample, leaving a continuous PME response close to T_C (Fig. 4e).

4) Superfluid density of a bulk superconductor

We obtain superfluid density of bulk Bi2212 by fitting susceptibility approach curves at each temperature point (Fig. S10a). In a strong bulk diamagnetism case, the sSQUID susceptibility takes the form $\frac{\phi(z)}{\phi_s} = -\frac{1}{[1+4(z/a+h_{eff}/a)^2]^{3/2}}$ [37]. $h_{eff} = z_0 + \lambda$ is taken as a fitting parameter, where λ is London penetration depth and z_0 is the remaining distance between the center of the pickup coil and the sample when the tip of the nano-SQUID touches the sample. For an alignment angle of 10 degrees, $z_0 = 1.3$ µm. Superfluid density is reciprocal to the square of penetration depth $\rho_s = \lambda^2(0)/\lambda^2(T)$. The zero-temperature intercept is taken as $\lambda(0)$.

In the temperature region away and below the transition, λ is about 100 nm in the bulk Bi2212 sample. We note that the height uncertainty is about 80 nm as we showed earlier (Fig. S3), which is on the same order as λ. This leads to a large uncertainty in ρ_s for this temperature range (Fig. S10b). Although keeping a light contact between the nano-SQUID and the sample may remove the uncertainty in z_0 [67], such thermal contact will significantly affect the SQUID operation at elevated temperatures. Nevertheless, the temperature dependence of ρ_s we obtain away from the transition is consistent within error with the DC susceptibility measured by vibrating sample magnetometry at 3 Oe (Fig. S10b, orange).

In the transition regime, on the other hand, λ is much bigger than the height uncertainty. Therefore, the error bars on ρ_s becomes significantly smaller than those in the low temperature regime (Fig. S10b). As a result, the sharp rise in ρ_s at T_C is well above the uncertainty level. In

contrast, bulk DC susceptibility measured by vibrating sample magnetometry does not exhibit such a sharp rise. Also absent is the small kink above the step. This suggests that the magnetic response integrated over phase domains by volumetric probes are not sensitive to the intra- and interlayer phase correlation within a single domain in bulk Bi2212.

5) $NbSe_2$ multilayer and bulk sample

First, we show that $NbSe_2$ multilayer does not exhibit any paramagnetic Meissner effect. In the magnetometry image taken at 5 K (Fig. S11a), we can see isolated vortices in this 10 nm thick flake sample. The susceptometry image (Fig. S11b) shows similar amplitude of diamagnetism as a quadruple-layer Bi2212 at low temperature (Fig. S8b). This is consistent with the fact that the bulk $\lambda = 190$ nm of $NbSe_2$ is close to that of optimally-doped Bi2212. The $\chi'(H, T)$ obtained in the middle of this sample over a similar reduced temperature and field range (Fig. S11c) as that of Bi2212 multilayers shows no paramagnetic peaks. On the other hand, a bulk flake sample (thickness > 200 nm) shows no continuous paramagnetic features at finite H as seen in bulk of Bi2212 (Fig. S11d). The zero-field $\chi'(T)$ of this thick $NbSe_2$ sample is shown in Figure 4f.

3. Simulation of the vortex dynamics using the Coulomb gas model

A Coulomb gas model is defined as the system consisting of particles which have equal magnitude of positive or negative charge. The two-dimensional Coulomb gas model can realize famous Berezinskii–Kosterlitz–Thouless (BKT) phase transition, and one can apply the model to the superconductor film [56]. In this appendix, we introduce the theoretical analytic method used here. Sec.1 describes how to map the two-dimensional superconductor to the 2D Coulomb gas. The major properties of the two-dimensional Coulomb gas are discussed in Sec.2. In Sec.3, we

introduce the boundary condition used in the calculation. Sec.4 contains the steps of the analytic method. Sec.5 is devoted to the discussion.

1) Mapping the two-dimensional superconductor to the two-dimensional Coulomb gas

We begin by giving a heuristic argument for the analogy between the two-dimensional superconductor and the 2D Coulomb gas [56]. In the following reasoning, we select some formulas from Ginzburg-Landau theory, and for more details, readers can refer to the standard textbook such as [62]. According to Ginzburg-Landau theory, the superconductor film state can be characterized by a complex order parameter: $\psi=\sqrt{\rho(\vec{r})}\,\mathrm{e}^{\mathrm{i}\theta(\vec{r})}$, where $\rho(\vec{r})$ is the superfluid mass density, which we approximate to a constant here, and θ is connected to the superfluid velocity $\vec{v}_s$ by,

$$\vec{v}_s(\vec{r})=\frac{\hbar}{m^*}\nabla\theta(\vec{r})-\frac{e^*\vec{A}}{m^*c}, \tag{1}$$

where $\hbar$ is reduced Planck constant, $m^*=2m_e$ and $e^*=2e$ are the mass and charge of a Cooper pair, $\vec{A}$ is gauge vector potential, and c is the speed of light. The energy of the superconductor is proportional to the superfluid velocity squared, i.e. $E^{SF}\propto v_s^2$. The thermal excitations in the superconductor film are vortices, and the integral around a closed loop surrounding vortices of the gradient of the phase $\theta(\vec{r})$ should always be integer multiples of 2π because of the singlevaluedness of ψ,

$$\int \mathrm{d}\,\vec{s}\cdot\nabla\theta(\vec{r})=2n\pi, \tag{2}$$

where n is an integer. Similarly, the charges in the 2D Coulomb gas can excite the electric field, the energy of the electric field is proportional to the electric intensity $\vec{E}$ squared, i.e. $E^{CG}\propto E^2$, and according to the Gauss's theorem, the integral around a closed surface surrounding charged particles of the electric intensity $\vec{E}$ is,

$$\int \mathrm{d}\vec{S}\cdot\vec{E}=2\pi q, \tag{3}$$

where q is the total charges enclosed by the integral surface. We can set q to be an integer by choosing electrostatic units. Therefore, we can analogize the vortex and the superfluid velocity in the superconductor film to the charged particle and the electric intensity in the Coulomb gas, respectively, except that the electric intensity is perpendicular to the superfluid velocity, namely, $\vec{E} \perp \vec{v}_s$ (Fig. S12a).

Now we derive the relation between the two-dimensional superconductor and the 2D Coulomb gas. The relation has been systematically demonstrated in the reference [56], and we outline the main steps of the demonstration here. The Hamiltonian of the system may be expressed as,

$$H_s = \int \mathrm{d}^2\vec{r} \left(\frac{\vec{g}^2(\vec{r})}{2\rho} + \frac{e^* \vec{A} \cdot \vec{g}(\vec{r})}{2m^* c} \right), \tag{4}$$

where $\vec{g} = \rho(\vec{r})\,\vec{v}_s(\vec{r})$ is the mass current density. Because of considering here how the fluctuations in the superfluid mass current influence the superfluidity, the mass current can be related to a scaler field Φ by,

$$\vec{g}(\vec{r}) = \nabla \times (\Phi(\vec{r})\vec{x}_3) - \frac{\rho e^* \vec{A}}{m^* c}, \tag{5}$$

where the unit vector $\vec{x}_3$ is perpendicular to the plane of the superconductor film. Substituting Eq. (1) and Eq. (5) into Eq. (2), with the help of the Stokes theorem, we finally get the 2D Poisson's equation,

$$\nabla^2 \Phi(\vec{r}) = -2\pi n \frac{\rho \hbar}{m^*} \delta(\vec{r}). \tag{6}$$

The two-dimensional Poisson's equation can be used to describe the system consisting of particles which have equal magnitude of positive or negative charges, which is usually called “the 2D Coulomb gas model”. Therefore, the vortex excited in the superconductor can be recognized as the charged particle in the Coulomb gas, and the scaler field Φ can be recognized as the electrostatic potential. The electric intensity $\vec{E}$ is defined as,

$$\vec{E} = -\nabla \Phi, \tag{7}$$

and the electric current density is defined as,

$$\vec{j} = \frac{e^*}{m^*}\vec{g}. \tag{8}$$

According to Eq. (1) and Eq. (2), the electric intensity is perpendicular to the electric current density, i.e. $\vec{E} \perp \vec{j}$.

2) The two-dimensional Coulomb gas model

In this section, we introduce the basic properties of the two-dimensional Coulomb gas.

A Coulomb gas model is defined as the system consisting of particles which have equal magnitude of positive or negative charge. The particles interact with each other through the two-dimensional Coulomb interaction, described by Poisson's equation,

$$\nabla^2 \varphi(\vec{r}) = -2\pi\delta(\vec{r}), \tag{9}$$

where $\varphi(\vec{r})$ is called the two-dimensional Coulomb potential. In two dimensions, by solving Eq. (9), the Coulomb potential, induced by a point charge q_i, has logarithmic dependence on the distance $\vec{r}$ to the charge,

$$\varphi_i(\vec{r}) = -q_i \ln(\vec{r}). \tag{10}$$

However, the divergence of the logarithmic function at $\vec{r} = 0, \infty$ is troublesome. To eliminate the singularity, one usually introduces the ultraviolet cutoff σ [63] [64] and infrared cutoff λ [65] for the Coulomb potential $\varphi_i(\vec{r})$, corresponding to the vortex core size and the system size for the 2D superconductivity, respectively,

$$\varphi_i(\vec{r}) = -q_i \ln\left(\frac{\vec{r}}{\lambda}\right), \tag{11}$$

which means that the distance between the particles can't be less than σ, and the interaction between the particles will be screened if the distance is more than λ. In this case, the self-energy E_0 of the point charge q_i can be expressed as,

$$E_0 = -q_i^2 \ln\left(\frac{\sigma}{\lambda}\right). \tag{12}$$

For many-particle systems, the total potential $\varphi(\vec{r})$ at the position $\vec{r}$ is,

$$\varphi(\vec{r}) = \sum_i \varphi_i(\vec{r}), \tag{13}$$

where the sum i is over all particles of the system.

Consider the Hamiltonian of the Coulomb gas system with fluctuations in the number of particles. We denote the number of particles with positive charges, negative charges, and any charges of the system as N^+, N^-, and N, respectively. The Hamiltonian of the system can be divided into electrostatic energy and nonelectrostatic energy parts [56]. The nonelectrostatic energy part, defined as the chemical potential of the positive (negative) charges $\mu^{+(-)}$, containing the core energy E_c and the difference in excitations for positive and negative charges, denoted by ΔE, can be expressed as [56],

$$\mu^{\pm} = -(E_c \pm \Delta E). \tag{14}$$

The relation between ΔE and the external magnetic field B for the 2D superconductivity is [56],

$$\Delta E = \frac{2\pi R^2}{\phi_0} B, \tag{15}$$

where ϕ_0 is the flux quantum. The electrostatic part W_N, i.e., the interaction energy of N particles, according to standard electrodynamics, has the following form,

$$W_N = \frac{1}{2} \iint \vec{E} \cdot \vec{D} \, \mathrm{d}S, \tag{16}$$

where $\vec{E}$ is the electric intensity, and $\vec{D}$ is the electric displacement. To simplify calculation, the Coulomb potential $\varphi(\vec{r})$, namely, Eq. (13), can be used, by the relation to the electric intensity,

$$\vec{E} = -\nabla\varphi. \tag{17}$$

Substituting Eq. (17) to Eq. (16), the electrostatic configuration energy can be expressed,

$$\begin{aligned} W_N &= -\frac{1}{2}\iint \nabla\varphi \cdot \vec{D}\,\mathrm{d}S \\ &= -\frac{1}{2}\iint \nabla\cdot\left(\varphi\vec{D}\right)\mathrm{d}S + \frac{1}{2}\iint \varphi\nabla\cdot\vec{D}\,\mathrm{d}S \\ &= -\frac{1}{2}\oint_b \varphi\vec{D}\cdot\mathrm{d}\vec{l} + \frac{1}{2}\iint \varphi\rho\,\mathrm{d}S, \end{aligned} \tag{18}$$

where the Gauss's theorem and the Maxwell's equation $\nabla\cdot\vec{D}=\rho$ are used, and ρ is the free charge density. Because of the conductor boundary of the system (see section 1.3), the charges are distributed on the surface, and their potential is equal, denoted φ_c. Therefore, we can further simplify Eq. (18) into,

$$\begin{aligned} W_N &= -\frac{1}{2}\oint_b \varphi\vec{D}\cdot\mathrm{d}\vec{l} + \frac{1}{2}\iint \varphi\rho\,\mathrm{d}S \\ &= -\frac{1}{2}\varphi_c\oint_b \vec{D}\cdot\mathrm{d}\vec{l} + \frac{1}{2}\iint (\varphi' + \varphi_c)\rho\,\mathrm{d}S \\ &= -\frac{1}{2}\varphi_c Q + \frac{1}{2}\sum_{i=1}^{N}\varphi_i' Q_i + \frac{1}{2}\varphi_c Q \\ &= \frac{1}{2}\sum_{i=1}^{N}\varphi_i Q_i, \end{aligned} \tag{19}$$

where the Gauss law $\oint_b \vec{D}\cdot\mathrm{d}\vec{l} = Q$ is used, and Q are total charges, namely, $Q=\sum_{i=1}^{N} Q_i$.

Substituting Eq. (13) into Eq. (19), we can get,

$$W_N = \frac{1}{2}\sum_{i=1}^{N} Q_i\left(\sum_{j=1}^{N}\varphi_j(\vec{r}_i)\right), \tag{20}$$

noting that $q_i\varphi_i(\vec{r}_i)$ should be replaced by the self-energy E_0 using Eq. (12). To sum up, the Hamiltonian of the system consisting of N particles is,

$$H = W_N - N^+\mu^+ - N^-\mu^-. \tag{21}$$

Next, we study the thermodynamic properties of the two-dimensional Coulomb gas model. The thermodynamic properties of the system are determined by its partition function Z, which is defined as,

$$Z = \sum_i \int \mathrm{d}\vec{r}_i \frac{\mathrm{e}^{-\beta H}}{N_i^+! N_i^-!}, \tag{22}$$

where $\beta = \frac{1}{T}$ is the temperature of the system. Substituting Eq. (14) and Eq. (21), the partition function Z is,

$$Z = \sum_{i=1}^{N} \int \mathrm{d}\vec{r}_i \frac{\mathrm{e}^{-\beta N_i^+(E_c + \Delta E)} \mathrm{e}^{-\beta N_i^-(E_c - \Delta E)}}{N_i^+! N_i^-!} \mathrm{e}^{-\beta W_i}. \tag{23}$$

In the remaining appendix, we focus on the partition function Eq. (23) to gain the thermodynamic properties of the model.

3) The boundary condition

The choice of boundary condition here for the two-dimensional Coulomb gas is that the system has the conductor boundary. For the superconductor film, the electric current can't cross its boundary. In other words, the transverse part of the electric current density is equal to zero on the walls of the superconductor film,

$$\vec{j}_\perp(boundary) = 0. \tag{24}$$

Because of the $\vec{E} \perp \vec{j}$, the boundary condition corresponds to the 2D Coulomb gas is that of the longitudinal part of the electric intensity zero on the walls,

$$\vec{E}_\parallel(boundary) = 0, \tag{25}$$

which means that the model has the conductor boundary. Therefore, the potential φ on the boundary is constant according to electrostatics,

$$\varphi(boundary) = constant. \tag{26}$$

We deal with the influence of the boundary on the system by introducing mirror charges. According to the uniqueness theorem for Poisson's equation, the boundary effect is equivalent to so-called "mirror charges" only if they satisfy Poisson's equation and the same boundary condition. Mirror charges q_i' must lie beyond the boundary, and are opposite to the original

charges q_i. What's more, the potential $\varphi_i'(\vec{r})$ induced by a mirror charge has the same form as Eq. (11). Therefore, more comprehensively, the total potential $\varphi(\vec{r})$ can be expressed as,

$$\varphi(\vec{r}) = \sum_i \varphi_i(\vec{r}) + \sum_{i'} \varphi_i'(\vec{r}), \tag{27}$$

where the second term is mirror charges contribution. The position and quantity of mirror charges depend on the shape of the boundary. For the circular boundary of radius R, setting the center of the circle O as the origin, the position $\vec{r}_i'$ and quantity q_i' of mirror charges for the 2D Coulomb gas are,

$$\begin{aligned} q_i' &= q_i, \\ r_i r_i' &= R^2, \end{aligned} \tag{28}$$

where q_i and $\vec{r}_i$ are the position and quantity of original charges, and $\vec{r}_i'$ is parallel to $\vec{r}_i$ (Fig. S12b).

4) The analytic method

In this section, we discuss the analytic method used here. We can obtain the thermodynamic properties of this two-dimensional Coulomb gas model by calculating the partition function Eq. (23). However, Eq. (23) is too complex to gain the exact solution by calculating it analytically. One can also get the numerical solution of Eq. (23) by Monte Carlo methods [66]. Here, we adopt a simpler scheme described below to calculate Eq. (23). The partition function Eq. (23) contains infinite sums over the number of particles so that we can't solve it directly. If the state of the system isn't close to the critical point, we can regard the fluctuations in the number of particles as the renormalization for the dielectric function ε [56]. In other words, the fluctuations can lead to the screening effect on the electric interactions between the particles, which can be described by ε. From the perspective of the renormalization group, we can define an effective temperature as,

$$\beta^* = \frac{1}{T\varepsilon}, \tag{29}$$

where T is the temperature of the system. Therefore, we can keep only the first few terms (such as the first four terms) related to the particles with negative charges to calculate the partition

function Z effortlessly. The dielectric function values at different temperatures can be obtained from the experiment.

We introduce the main procedures of the method as follows. In the following, we set the circular boundary of the system, and we chose units such that the charges of the particles $q_i = \pm 1$. Firstly, we express the partition function Z as,

$$\begin{aligned} Z = 1 + C_1 \mathrm{e}^{-\beta^*(E_c - \Delta E)} + \frac{C_2}{2}\mathrm{e}^{-2\beta^*(E_c - \Delta E)} \\ + \frac{C_3}{6}\mathrm{e}^{-3\beta^*(E_c - \Delta E)} + \frac{C_4}{24}\mathrm{e}^{-4\beta^*(E_c - \Delta E)}. \end{aligned} \tag{30}$$

The coefficients C_1, C_2, C_3, and C_4 are the following integrals,

$$\begin{aligned} C_N &= \int \mathrm{d}\vec{r}\,\mathrm{e}^{-\beta^* W_N} \\ &= \int \mathrm{d}\vec{r}\,\mathrm{e}^{-\beta^* \frac{1}{2}\sum_{i=1}^{N} q_i \varphi(\vec{r}_i)} \\ &= \int \mathrm{d}\vec{r}\,\mathrm{e}^{-\beta^* \frac{1}{2}\sum_{i=1}^{N} q_i \left(\sum_{j=1}^{N} -q_j \ln\left(\frac{\vec{r}_i - \vec{r}_j}{\lambda}\right) + \sum_{j=1}^{N} -q_j{}' \ln\left(\frac{\vec{r}_i - \vec{r}_j{}'}{\lambda}\right)\right)}, \\ &(N = 1, 2, 3), \end{aligned} \tag{31}$$

where we use Eq. (11), Eq. (23), Eq. (20), Eq. (27) and Eq. (29). We calculate the coefficients with Monte Carlo methods. Secondly, solve the free energy of the system taking advantage of Z ,

$$F = -\frac{1}{\beta^*}\ln Z. \tag{32}$$

Finally, extract the magnetic susceptibility χ from the free energy according to thermodynamics,

$$\chi = -\frac{\partial^2 F}{\partial B^2}, \tag{33}$$

where B is the external magnetic field of the system. Substituting Eq. (15) to Eq. (33), the magnetic susceptibility χ is,

$$\chi = \frac{\partial^2 F}{\partial \Delta E^2}\frac{\partial^2 \Delta E}{\partial B^2}. \tag{34}$$

Plotting the magnetic susceptibility χ as a function of magnetic field B, we can get the results Fig. 3c and Fig. S12 in the supplementary III. In this calculation, E_c and R, introduced in Sec. 2, are treated as free fitting parameters and obtained by fitting the location of the peaks to experimental data.

5) Discussion

It is well known that the two-dimensional Coulomb gas model undergoes Berezinskii–Kosterlitz–Thouless (BKT) phase transition when varying the temperature T [56], and we can also see that from the results Fig. 3c in the body and Fig. S12 in the supplementary III. Below the BKT critical temperature T_c, the model belongs to a bound dipole phase (i.e. vortex forming dipoles), in which the model has a finite value of the dielectric function ε. Above T_c, the model realizes a plasma phase, in which the dipoles dissociate and the dielectric function ε is infinite, resulting in the interaction between the charges are completely screened. For the results Fig. 3c and Fig. S12, the peaks of the curve for the susceptibility χ versus the external magnetic field B is related to the excited vortex, and the width of the peaks is associated with the thermal fluctuation of the vortex, described by the effective temperature β^* in Eq. (29). Therefore, below T_c, because of finite dielectric function ε leading to finite thermal fluctuation β^*, we can see different peaks with finite width corresponding to some excited vortex in the results. While above T_c, infinite ε results in the width of the peaks infinite so that we can not distinguish them.

References for the Supplementary Materials

[62] L. D. Landau and E. M. Lifshitz, *Statistical physics*, v. 5. Course of Theoretical Physics, 23, 1980.

[63] S. Samuel, *Grand partition function in field theory with applications to sine-gordon field theory*, Physical Review D, 18(6):1916, 1978.
[64] P. Minnhagen, A Rosengren, and G Grinstein, *Screening properties of a classical two-dimensional coulomb gas from the sine-gordon equation*, Physical Review B, 18(3):1356, 1978.
[65] P. Minnhagen, *Kosterlitz-thouless transition for a two-dimensional superconductor: Magnetic-field dependence from a coulomb-gas analogy*, Physical Review B, 23(11):5745, 1981.
[66] J. Lidmar and M. Wallin, *Monte carlo simulation of a two-dimensional continuum coulomb gas*, Physical Review B, 55(1):522, 1997.
[67] C. Hicks, T. Lippman, M. Huber, J. Analytis, J.-H. Chu, A. Erickson, I. Fisher, and K. Moler, *Evidence for a Nodal Energy Gap in the Iron-Pnictide Superconductor LaFePO from Penetration Depth Measurements by Scanning SQUID Susceptometry*, Phys. Rev. Lett. **103**, 127003 (2009).

III. Figures S1 to S12

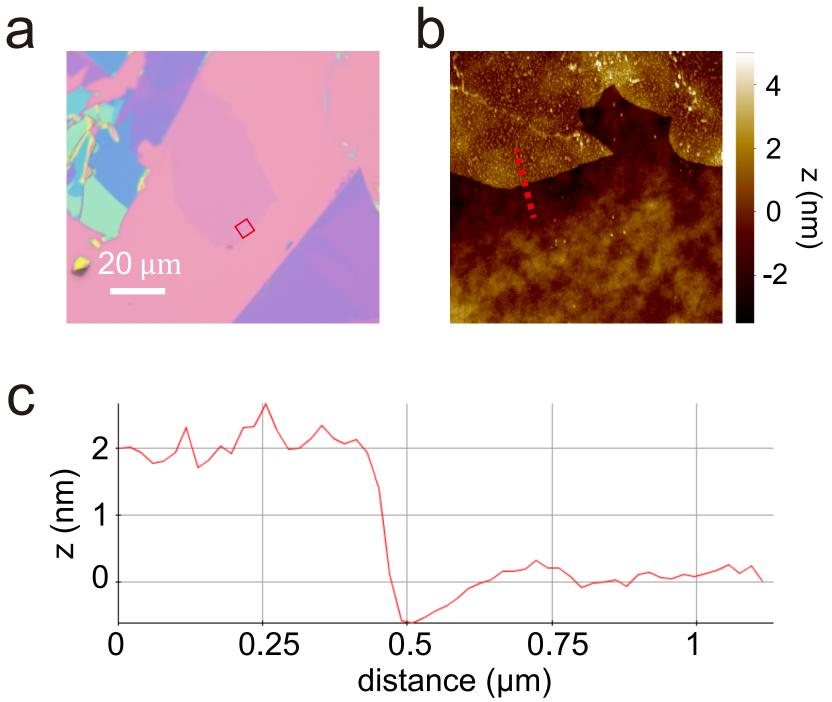

Fig. S1 Atomic force microscopy (AFM) image of a monolayer sample. **a**, Optical image of the sample. **b,** AFM image of the red box area in **a**. **c**, Line cut across the edge of the monolayer as labeled by the red line in **b**.

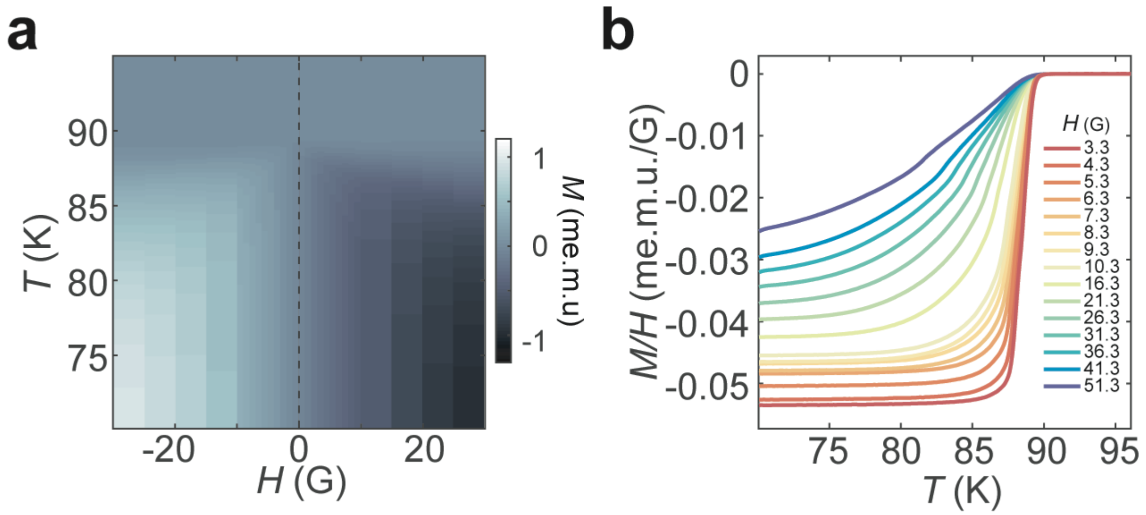

Fig. S2 Vibrating sample magnetometry of a bulk Bi2212 sample. a, Magnetization (*M*) as a function of temperature (*T*) and external perpendicular field (*H*). **b,** DC susceptibility as a function of *T* at various *H* obtained from **a** by *M*/*H*.

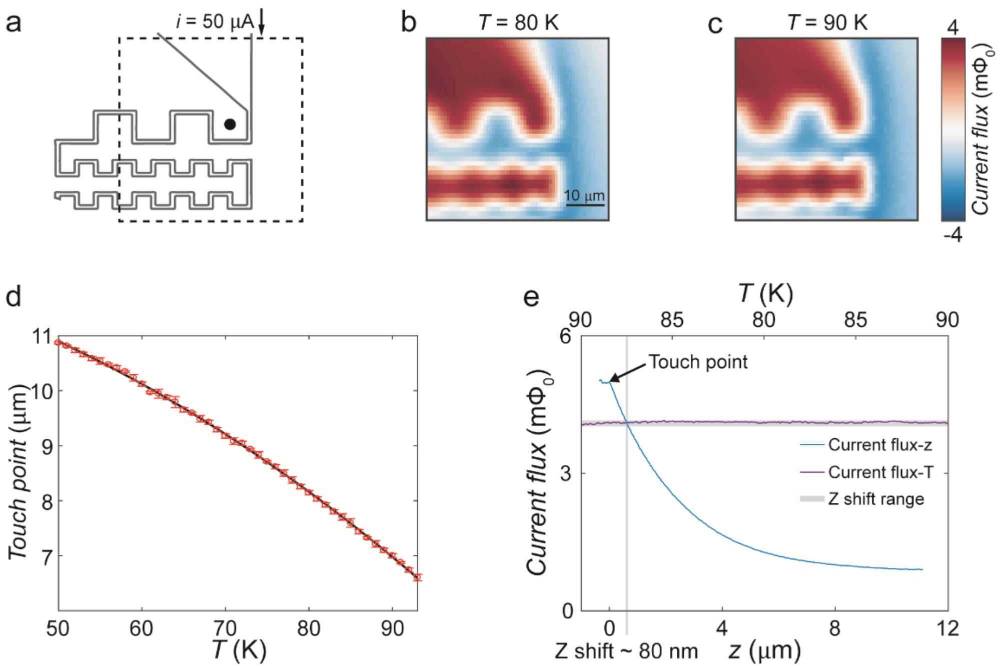

Fig. S3 Sample displacement calibration for variable temperature scanning SQUID. **a,** Current pattern for displacement calibration. A 50 μA AC current is applied through a meandering Nb wire to simulate AC magnetic response. The black point is the position for fixed point measurements. **b** and **c**, Current flux images obtained at T = 80 K (b) and T = 90 K (c) respectively, around the dashed region outlined in **a**. There are no notable difference in the meander region, suggesting no distinguishable in plane shift under our spatial resolution. **d**, Approach points measured as a function of temperature by a capacitive sensor the nano-SQUID is mounted on. Approach curves are performed repeatedly for 5 times after the temperature has stabilized at each point. The 'touch point' is the average of the 5 approach points. The black line is a parabolic fit. **e**, Approach curve at T = 80 K (blue) and current flux versus T curve (purple) after compensating for the variation in touch point. The variation of current flux with changing temperature is cycled under a 90 K – 80 K – 90 K temperature sequence (top horizontal axis). If we assume the SQUID signal is not directly affected by temperature, the variation of the current flux signal under the temperature sequence corresponded to about 80 nm of height variation.

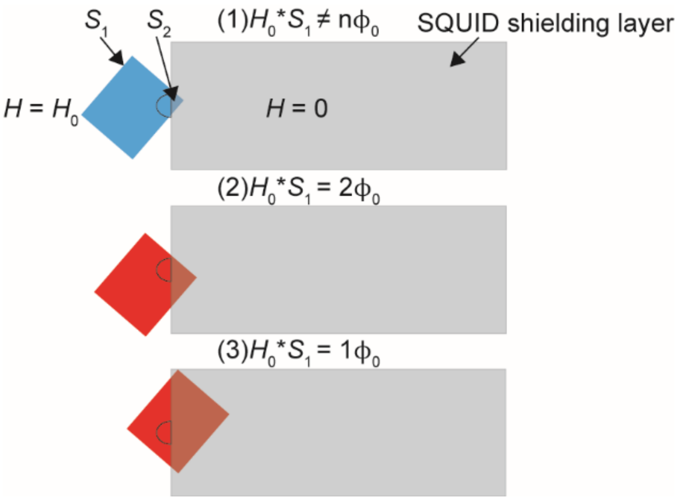

Fig. S4 A sketch of the relative position between nano-SQUID and sample during scanning. The hollow semicircle represents the SQUID's pickup loop and grey rectangular is the magnetic shielding layer on the nano-SQUID chip. The diamond shape pattern on the left beside the SQUID is the sample domain with an area of $S_0 = S_1 + S_2$, where S_1 is the space outside the shielding layer and S_2 is inside the shielding layer. The color of the domain represents whether the domain is diamagnetic (blue) or paramagnetic (red).

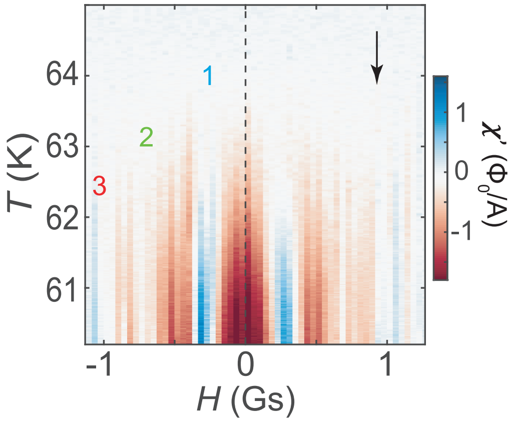

Fig. S5 Oscillating paramagnetic susceptibility in temperature sweeps of the monolayer sample. The data is obtained by fixing the field H and repeatedly cooling the sample through the transition temperature. The measurement location on the monolayer is labeled in Fig. 2b (the same location as that of the data in Fig. 3a). There is no interpolation to generate the figure.

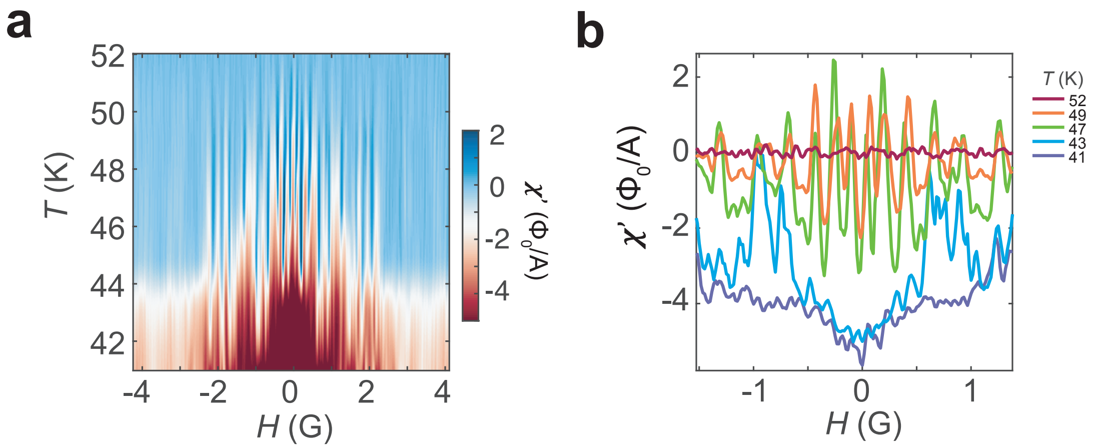

Fig. S6 Oscillating paramagnetic susceptibility of the monolayer sample at a different sample location. The measurement location is at the blue dot labeled in Fig. 2b**. a,** $\chi(T,H)'$ obtained by sweeping field at fixed temperature. The T axis is interpolated to generate the image. **b,** $\chi(H)'$ at several T obtained from **a**. $T_\mathrm{C} = 52$ K at this domain.

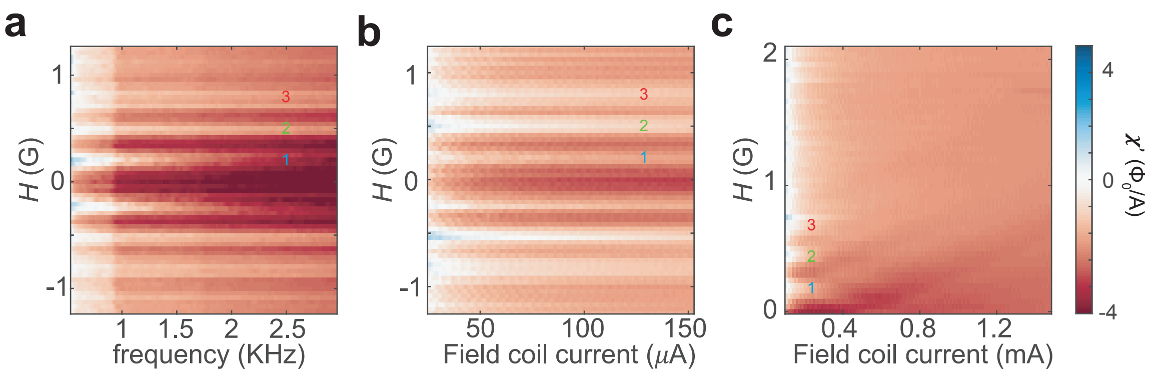

Fig. S7 Changing frequency and amplitude of the field coil modulation current. a, Susceptibility as a function of the alternating-current frequency through the field coil at various *H*. **b** and **c,** Susceptibility as a function of the driving amplitude through the field coil at various *H* for two different range of the amplitude, respectively. All the data are obtained at 60 K at the location on the monolayer labeled in **Fig. 1h**. The paramagnetic peaks stay constant with the driving frequency or the amplitude when the amplitude is small.

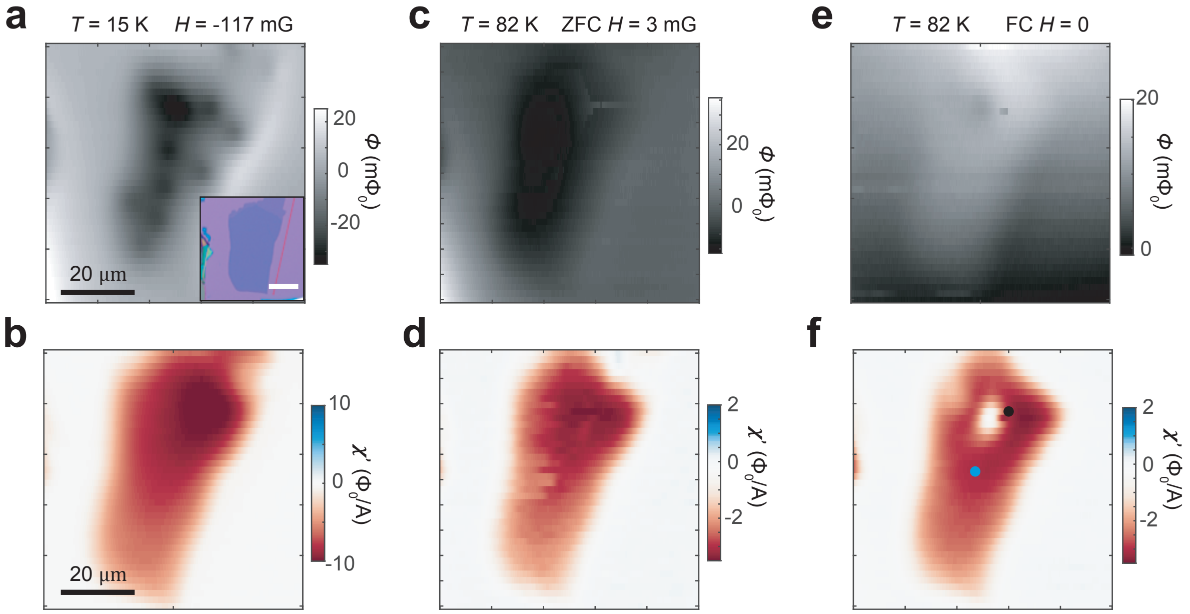

Fig. S8 sSQUID images of the Bi2212 quadruple-layer sample. a and **b**, Magnetometry and susceptometry images at $T = 15$ K and $H = -117$ mG. Inset: optical image of the sample. The white scale bar is 20 μm. **c** and **d**, Magnetometry and susceptometry images at $T = 82$ K and $H = 3$ mG after zero-field cooling. **e** and **f**, Magnetometry and susceptometry images at $T = 82$ K and $H = 0$. The sample is cooled under a small field so that a single vortex is trapped in the upper part of the sample. Data shown in Figs. 4**a** and **b** are obtained at the black point in **f**.

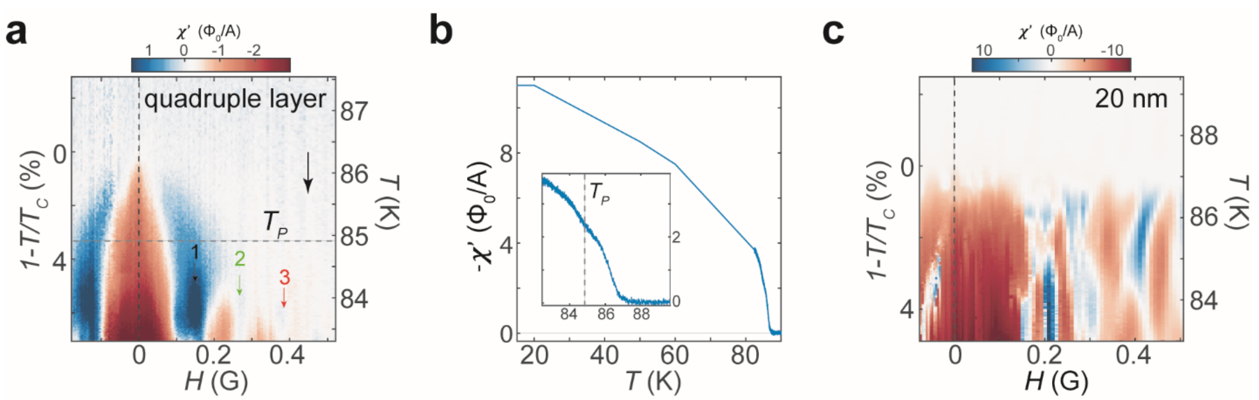

Fig. S9 Oscillating paramagnetic Meissner effect of the multilayer samples. a, $\chi(T,H)'$ of the quadruple layer sample obtained at the blue point shown in Fig. S8f. The T_C of this area is slightly lower than that of the one shown in Figure 4. The gray dashed line at T_P separates the transition into two temperature regimes. For $T_\mathrm{P} < T < T_\mathrm{C}$, there is no oscillation of the PME. **b,** $-\chi(T)'$ at zero-field. The high temperature regime ($T > 83$ K) is obtained from **a** and the rest part is obtained at several temperature points without varying the field. Inset: a zoom in of the high temperature regime. The kink at 85 K is also visible at this location. **c,** $\chi(T,H)'$ of a 20 nm sample showing more sporadic PME behavior.

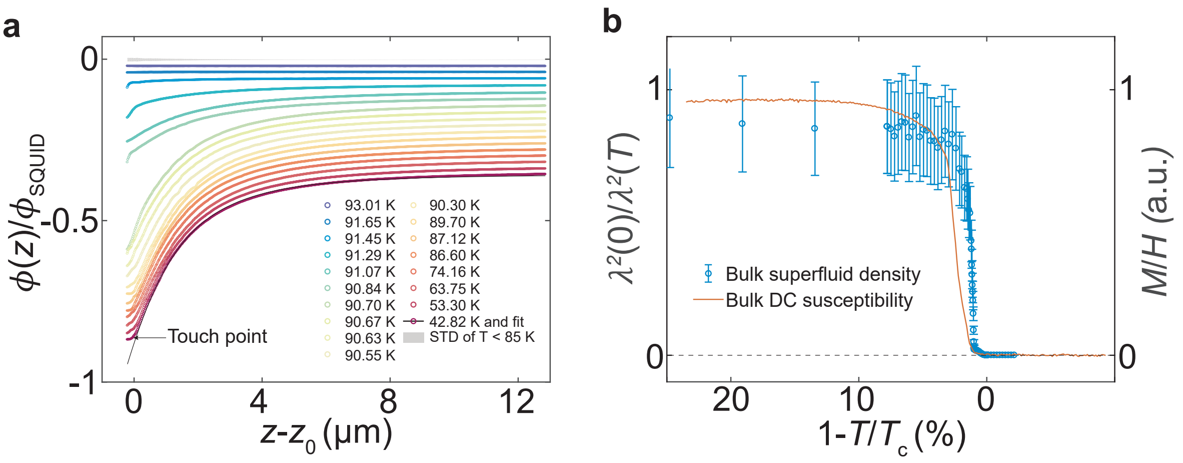

Fig. S10 Bulk Bi2212 superfluid density. a, Susceptibility as a function of nano-SQUID height z at various representative temperatures, which are used for extracting the bulk superfluid density in Fig. 4f. The approach point z_0 is determined by a capacitive sensor the nano-SQUID mounted on. The fitting (see text) at $T = 42.82$ K shows a good match between our approach curve and a bulk diamagnetism model. STD is the standard deviation of curves at $T < 85$ K. **b**, Comparison between $\lambda^2(0)/\lambda^2(T)$ measured by sSQUID at zero field and DC susceptibility M/H measured by vibrating sample magnetometry at 3 Oe of a bulk sample. Only $\lambda^2(0)/\lambda^2(\mathrm{T})$ shows the kink near $T_\mathrm{C} = 91.5$ K. And the jump in $\lambda^2(0)/\lambda^2(\mathrm{T})$ is steeper than that in the DC susceptibility curve.

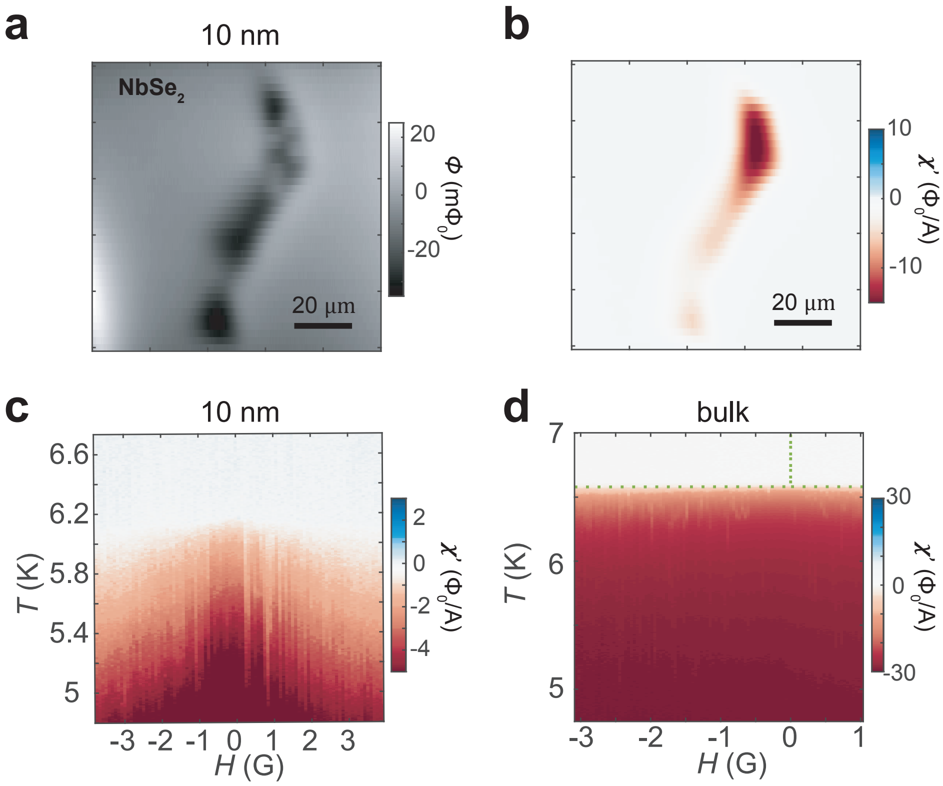

Fig. S11 sSQUID images of $NbSe_2$ samples. **a** and **b,** flux and χ' image of a 10-nm-thick $NbSe_2$ flake at 5 K. **c** and **d,** χ' of the 10-nm $NbSe_2$ flake and a bulk sample (at least 200 nm thick) as a function of *T* and *H* obtained by sweeping temperature at fixed field. The $\chi'(T)$ at zero-field of the bulk sample is shown in Fig. 4**f**. Even though the London penetration depth and coherence length of $NbSe_2$ are close to that of Bi2212, there are no observable paramagnetic susceptibility over a similar $\frac{T}{T_C} - H$ parameter space.

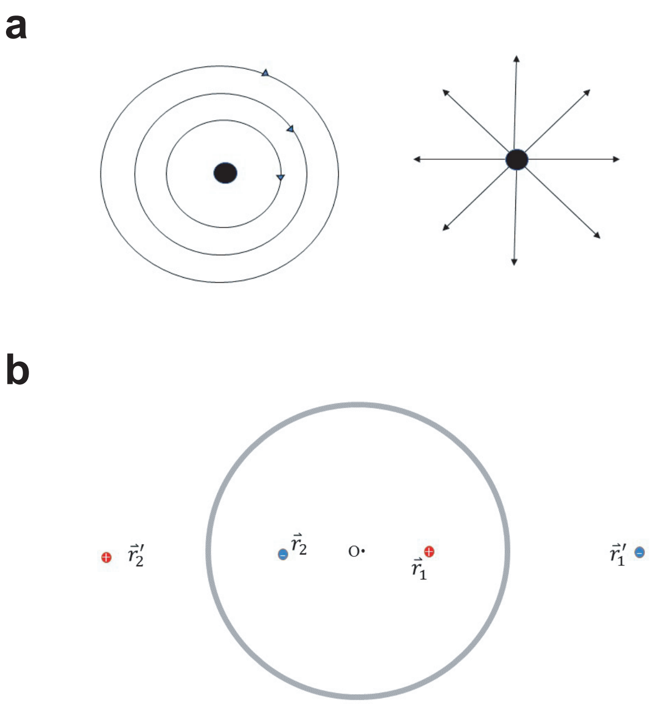

Fig. S12 Illustration of the Coulomb gas model. a, Sketch of the duality between the electric current density of a vortex (left) and the electric field produced by a charge (right). **b,** Sketch of mirror particles for a circular geometry. The grey circle is the sample boundary.
